# The role of sea fish meat in the transmission of *Vibrio parahaemolyticus* to humans: An in-depth analysis of seasonal and species-specific variations

**DOI:** 10.14202/vetworld.2025.348-354

**Published:** 2025-02-17

**Authors:** Maged A. Al-Garadi, Dhary Alewy Almashhadany, Rasha N. Aziz, Dheyazan M. Ali Al-Qabili, Ohoud S. Alhumaidan, Hanouf Alnuwaysir, Al-Hammadi Mohammed Ali, Essam Sayed, A. M. Alabsi

**Affiliations:** 1 Department of Animal Production, College of Food and Agriculture Sciences, King Saud University, Riyadh, 1145, Kingdom of Saudi Arabia; 2 Department of Medical Laboratory Science, College of Science, Knowledge University, Erbil, 44001, Iraq; 3 Department of Public Health and Zoonoses, Faculty of Veterinary Medicine, Thamar University, Dhamar, Yemen; 4 Department of Clinical Laboratory Sciences, College of Applied Medical Sciences, King Saud University, Riyadh, 12372, Kingdom of Saudi Arabia; 5 Microbiology Unit in the Central Research Laboratory, King Saud University, Riyadh, Kingdom of Saudi Arabia; 6 Department of Microbiology, College of Veterinary Medicine, King Faisal University, Al-Ahsa, Kingdom of Saudi Arabia; 7 Special Study Office, Abu Dhabi Agriculture and Food Safety Authority, Abu Dhabi, P. O. Box., 71033, United Arab Emirates; 8 Department of Preclinical Sciences, School of Dentistry, Management and Science University, University Drive, off Persiaran Olahraga, 40100, Sha Alam, Selangor, Malaysia

**Keywords:** fish meat, foodborne infection, seafood safety, seasonal variation, *Vibrio parahaemolyticus*

## Abstract

**Background and Aim:**

*Vibrio parahaemolyticus* is a marine bacterium commonly associated with foodborne illnesses due to the consumption of contaminated seafood. Understanding its prevalence in both fish meat and human infections is crucial for public health. This study aimed to estimate the occurrence of *V. parahaemolyticus* in human stool and fish meat samples while analyzing seasonal and species-specific variations in the Al-Hodeidah governorate.

**Materials and Methods:**

A total of 225 samples were collected, including 75 human stool samples from patients with gastrointestinal symptoms and 150 fish meat samples from five fish species commonly consumed in the region. Standard microbiological methods were used for the isolation and identification of *V. parahaemolyticus*, including culture on Thiosulfate–Citrate–Bile Salts–Sucrose (TCBS) agar, biochemical tests, and growth analysis in varying NaCl concentrations. Data were statistically analyzed using SPSS version 12, applying the Chi-square test for group comparisons with a significance level of p ≤ 0.05.

**Results:**

The overall occurrence of *V. parahaemolyticus* was 7.1%. Human stool samples had a occurrence of 6.7%, while fish meat samples had a slightly higher occurrence of 7.3%. The highest monthly occurrence in human samples was recorded in July (15.0%), while the highest fish contamination was detected in September (12.0%). Among fish species, *Rastrelliger kanagurta* (Bagah) had the highest contamination rate (20.0%), followed by *Scomberomorus commerson* (Dairak) at 13.3%, whereas no *V. parahaemolyticus* isolates were found in *Dasyatis kuhlii* (Safon) and *Rachycentron canadum* (Sakalah).

**Conclusion:**

The findings confirm the presence of V. parahaemolyticus in both human and fish meat samples, highlighting seasonal variations and species-specific differences. The peak occurrence in fish during warm months suggests a potential link between higher temperatures and bacterial prevalence. Improved seafood handling, monitoring, and public health awareness are essential to mitigate the risk of foodborne infections. Further research is needed to explore genetic determinants of virulence and antimicrobial resistance in local isolates.

## INTRODUCTION

Today, the world is witnessing a worldwide increase in the consumption of fish meat because of the increased awareness about its low cholesterol and fat content, which is an important source of vitamins, minerals, polyunsaturated fatty acids, and high-quality animal protein content, making it an important component in the human diet [1]. Fish and seafood constitute an important food component for a large portion of the world population, followed by red meat and poultry as staple animal protein sources. In particular, fish are a cheap source of protein. However, seafood is prone to bacterial contamination, especially in filter feeders such as mussels, which concentrate bacteria in their filtration systems. Consequently, these filter feeders are ideally suited to trap all bacteria and viruses that are pathogenic or otherwise live in the water [2].

The occurrence of *Vibrio* species in raw seafood is common, particularly in regions with temperate climates around the world, encompassing both natural and farm environments, and affecting seafood of all types [2, 3]. *Vibrio parahaemolyticus*, a widely distributed Gram-negative curved rod found in marine environments, is a major cause of foodborne illness associated with the consumption of raw, undercooked, or contaminated seafood [4]. It can cause mild-to-moderate gastrointestinal infections, which are usually self-limiting and rarely fatal. The risk of infection is higher because these pathogens are resistant to most antibiotics. The pathogenicity factors of *V. parahaemolyticus* are attributed to the presence of thermostable direct hemolysin (*tdh*) and *tdh*-related hemolysin (trh) genes.

Many outbreaks of foodborne infection, especially in Asian countries, have frequently been reported to be due to the presence of *V. parahaemolyticus*. Although the incidence of *V. parahaemolyticus* infection is not as frequent as in Asia, several outbreaks have been reported in the United States and Europe [5]. *V. parahaemolyticus* was first identified by *T. Fujino* as a foodborne pathogen in Japan in 1951. By the late 1960s and early 1970s, *V. parahaemolyticus* had been recognized as a cause of diarrheal disease worldwide, although it was most common in Asia and the United States [6, 7].

Medically, *V. parahaemolyticus* is a leading cause of gastroenteritis worldwide, especially in coastal regions; however, the actual burden is likely to be much higher due to underreporting and diagnostic challenges [8]. The clinical manifestations of *V. parahaemolyticus* infection include watery diarrhea, abdominal cramps, nausea, vomiting, fever, and chills. Although most cases are self-limiting, severe infections can occur, especially in immunocompromised individuals, necessitating antibiotic treatment. However, the increasing prevalence of antimicrobial resistance in *V. parahaemolyticus* poses a significant challenge in the management of severe cases [4, 9].

The global dissemination of highly virulent and antimicrobial-resistant strains of *V. parahaemolyticus*, such as the O3:K6 serotype, poses a significant public health concern [10]. Climate change and rising sea temperatures have been implicated in the increased prevalence and geographic expansion of *V. parahaemolyticus*, further exacerbating the risk of foodborne outbreaks. Little is known regarding the prevalence of pathogenic *V. parahaemolyticus* in locally consumed fish. For a clear understanding of *V. parahaemolyticus* transmission, it is necessary to analyze the occurrence of *V. parahaemolyticus* in both human stool and fish meat, considering seasonal and species-specific variations often not adequately examined in similar studies. This study aimed to determine the occurrence of *V. parahaemolyticus* in human stool and sea fish meat samples in the Al-Hodeidah governorate, with a focus on seasonal variations and species-specific differences to better understand its transmission dynamics and public health implications.

## MATERIALS AND METHODS

### Ethical approval and Informed consent

The study protocol was reviewed and approved (No. 251220) by the Ethics Committee of the Public Health Department at the College of Veterinary Medicine, Thamar University, in accordance with international ethical standards for research involving human participants. Written informed consent was obtained from all participants before sample collection.

### Study period and location

The study was conducted from July 2023 to September 2023, during which a total of 225 samples were collected for analysis. Human stool samples were obtained from Al-Thawrah Hospital and various private medical laboratories, while fish meat samples were collected from five different fish species. All samples were collected in sterile conditions to ensure accuracy and reliability.

### Sample collection and preparation

Two hundred twenty-five samples were collected as follows: 75 human stools (44 male stool samples and 31 female stool samples), and 150 fish meat samples for five different species (30/each). The sample size was determined using the estimated prevalence of *V. parahaemolyticus* from the study of Al-Garadi *et al*. [11] to ensure sufficient power to detect significant differences. Human stool samples were collected if patients had gastrointestinal symptoms and consented to participate in the study. Fish meat samples were selected based on freshness, type, and availability in local markets to ensure a diverse representation of commonly consumed species.

Stool samples were collected from Al-Thawrah Hospital and other private medical laboratories in dry sterile containers, as described by Al-Garadi *et al*. [11] and Al-Mashhadany and Mayass [12]. Stool samples were directly plated on Thiosulfate–Citrate–Bile Salts–Sucrose (TCBS) selective agar plates (Himedia, India) and incubated for 24 h at 37°C. All fish meat samples were collected in sterile, labeled, sealed plastic bags before transport. Samples were analyzed immediately on the day of sampling according to the methodology described by Ahmad *et al*. [13]. Briefly, 10 g of fish meat was homogenized in 90 mL of alkaline saline peptone water (ASPW) in a sterile polythene stomacher bag for 1 min. Incubation of the first enrichment was performed at 41.5°C ± 1°C for 6 h ± 1 h, after which, a 10 mL volume of the first enrichment culture (taken from the surface of the broth) was transferred to 90 mL ASPW as the second enrichment broth. Subsequently, a loopful (1 µL) from the second enriched broth was streaked onto TCBS agar plates [14].

### Detection and identification of *V. parahaemolyticus*

Typical colonies of *V. parahaemolyticus* on TCBS appeared as dark bluish-green with 2–3 mm in diameter. Presumptive identification tests included: morphological shape, Gram staining, catalase, oxidase, urease, indole, motility, Methyl-Red-Voges-Proskauer, citrate utilization test, Triple Sugar Iron (Hi-Media), growth on nutrient broth at different NaCl concentrations (3%, 6%, 8%, and 10%), and lack of growth in the absence of NaCl. Biochemical tests were performed according to reference protocols [14].

### Statistical analysis

All data were analyzed using IBM SPSS Statistics version 21 (IBM, Armonk, NY, USA). Descriptive statistics were used to summarize the prevalence of *V. parahaemolyticu*s in human and fish meat samples. The Chi-square test (χ²) was applied to assess differences in prevalence rates between sample types, gender, fish species, and seasonal variations. A p-value of ≤ 0.05 was considered statistically significant.

The Wilson method was used to calculate 95% confidence intervals (CIs) for estimates, providing a more accurate measure of proportion estimates in small sample sizes. Additionally, binomial proportion estimates were applied to account for non-detected isolates in some fish species. All results are presented as percentages with corresponding CIs for better interpretability.

## RESULTS

### Occurrence of *V. parahaemolyticus*

**Figure 1:**
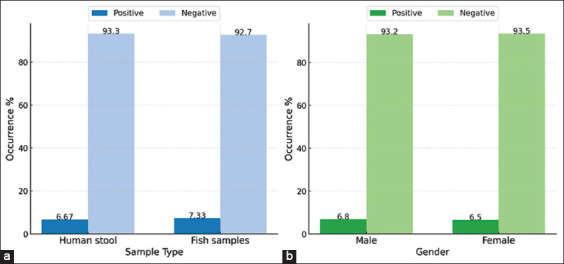
Overall occurrence of *Vibrio parahaemolyticus* in humans and fish in the Al-Hodeidah governorate. (a) Distribution of positive samples based on sample source. (b) Gender-based distribution of positive samples.

Two hundred twenty-five samples were collected in this study, 75 (33.33%) of which were human stool samples, and 150 were fish meat samples (66.67%). The overall percentage of *V. parahaemolyticus* in human and fish meat samples was 16 (7.1%) without any significant differences between the two groups (p = 0.869, χ^2^ = 0.027). The occurrence details are presented in Table 1 and summarized graphically in Figure 1. Gender seems not to play a role in infection rate, as no significant difference was found between males and females (Table 1).

**Table 1:** Overall occurrence of *Vibrio parahaemolyticus* in fish and human samples (n = 225).

Samples source	Number of samples	Positive, n (%)	95% CI	p-value
Sample types				
Human stool	75	5 (6.7)	2.88–14.68	0.869
Fish meat	150	11 (7.3)	4.14–12.65	
Total	225	16 (7.1)	4.42–11.24	
Sex				
Male	44	3 (6.8)	2.35–18.23	0.683
Female	31	2 (6.5)	1.79–20.72	
Total	75	5 (6.7)	2.88–14.68	

CI=Confidence interval

### The occurrence of *V. parahaemolyticus* according to months

**Figure 2:**
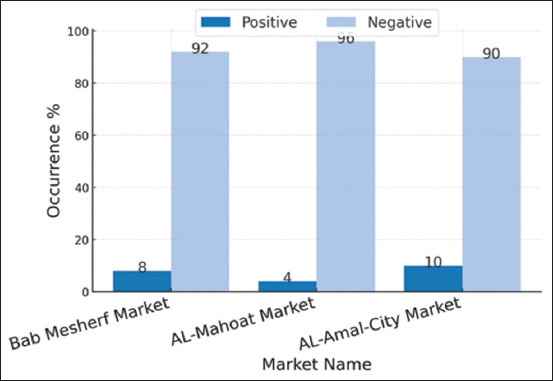
The occurrence of *Vibrio parahaemolyticus* in fish meat according to the area (market).

Regarding the temporal distribution of *V. parahaemolyticus*, the highest occurrence rates in fish and humans were recorded in September (12.0%) and July (15.0%), respectively (Table 2 and Figure 2). and the lower rate (2.9%) was recorded in June (Table 2). Statistical analysis showed no significant differences between months in the occurrence of *V. parahaemolyticus*.

**Table 2:** Monthly occurrence of *Vibrio parahaemolyticus* in fish and human stool samples.

Month	Number of samples	Positive samples, n (%)	95% CI	p-value
Fish meat				
July	50	3 (6.00)	2.06–16.22	0.283
August	50	2 (4.00)	1.10–13.46	
September	50	6 (12.00)	5.62–23.80	
Total	150	11 (7.33)	4.14–12.65	
Human stool				
July	20	3 (15.00)	5.24–36.04	0.412
August	20	1 (5.00)	0.89–23.61	
September	35	1 (2.86)	0.51–1.43	
Total	75	5 (6.67)	2.88–14.68	

CI=Confidence interval

### The occurrence of *V. parahaemolyticus* in fish meat

The results of the occurrence of *V. parahaemolyticus* in fish meat are presented in Table 3 and Figure 2. Statistically, there is no significant difference between markets in terms of *V. parahaemolyticus* occurrence (p = 0.509) despite the predicted 95% confidence intervals ranging from 1.10 to 13.46 for Al-Mahoat market to 4.35–21.36 for AL-Amal-City Market.

**Table 3:** The abundance of *Vibrio parahaemolyticus* in fish meat according to area (market).

Area	Number of samples	Positive, n (%)	95% CI	p-value
Bab Mesherf market in Pakistan	50	4 (8)	3.15–18.84	0.509
Al-Mahoat market	50	2 (4)	1.10–13.46	
AL-Amal-City market	50	5 (10)	4.35–21.36	
Total	150	11 (7.3)	4.14–12.65	

CI=Confidence interval

### The occurrence of *V. parahaemolyticus* in fish meat according to fish species

The overall occurrence rate of *V. parahaemolyticus* in different types of fish subjected to examination was 11/150 (7.3%) samples, indicating a significant difference between the analyzed fish species (Table 4). Although no isolate was recovered from two fish types (60 samples), this does not guarantee 100% certainty of the absence of *V. parahaemolyticus*, and prediction of an upper limit (11.35% for each type) is expected specifically for such a scenario of binomial proportion.

**Table 4:** The abundance of *Vibrio parahaemolyticus* in fish meat according to fish type.

Local name (scientific name)	Samples n	Positive, n (%)	95% CI	p-value
Safon (*Dasyatis kuhlii*)	30	0 (0.0)	0–11.35	0.001
Sakalah (*Rachycentron canadum*)	30	0 (0.0)	0–11.35	
Bagah (*Rastrelliger kanagurta*)	30	6 (20.0)	9.51–37.31	
Dairak (*Scomberomorus commerson*)	30	4 (13.3)	5.31–29.68	
Beath (*Flavocaranx bajad*)	30	1 (3.3)	059–16.67	
Total	150	11 (7.3)	4.14–12.65	

CI=Confidence interval

## DISCUSSION

*Vibrio* species are widespread in marine and estuarine environments, and several pathogenic species are known to be commonly associated with outbreaks of *Vibrio* infections due to consumption of raw fish or other seafood or through water contaminated with human feces or sewage [5, 6, 8]. *V. parahaemolyticus* is an important foodborne pathogen; therefore, it is essential to obtain data on the epidemiology, transmission, and control of this microorganism in human and fish meat for biosafety assessment [2, 3, 7]. Pathogenic vibrios are a public health concern for seafood consumers and are the cause of import bans in certain cases [15]. *V. parahaemolyticus* is a natural flora of estuarine and coastal marine environments worldwide. It has been isolated from sea and brackish water of both tropical and temperate regions [16, 17].

Samples of human stools and fish meat analyzed microbiologically in this study showed varying degrees of *V. parahaemolyticus* contamination. This study showed that 7.3% of isolates of *V. parahaemolyticus* were obtained from fish samples. These results are compatible with a similar study in Bulgaria [18], in which 6% of fish samples were reported to contain *V. parahaemolyticus*. However, higher rates of *V. parahaemolyticus* occurrence in fish and seafood samples were reported in different countries, including 11.1% in Nigeria [19], 12% in Jordan [20], 21.7% in Thailand [21], 23.4% in China [22], and 63.75% in Bangladesh [23].

Variations in the frequency of isolation of *V. parahaemolyticus* across studies can be attributed to differences in geographical regions, seasonality, sampling methods, laboratory techniques, fish species, sizes, environmental factors, human activities, and temporal trends. These factors influence bacterial prevalence through varying environmental conditions, water temperatures, sampling and processing methods, pollution levels, and human impact, leading to differing results in bacterial isolation rates [8, 15, 19].

The results of this study revealed that the occurrence rate of *V. parahaemolyticus* in human stool was 6.7 %. The results of this study are in contrast with the findings of published reports that the numbers of isolates and infection rates ranged from 25.0% to 29.0% [24, 25]. The prevalence of *V. parahaemolyticus* in asymptomatic humans is not well-documented due to the absence of systematic screening or investigation. However, studies have shown that *V. parahaemolyticus* can be present in clinically asymptomatic seafood workers, indicating that asymptomatic carriage is possible [26]. In terms of seasonality, the results showed that the occurrence *V. parahaemolyticus* in humans was higher in July and declined to a lower level in September. Statistically, no significant difference was observed between *the occurrence of V. parahaemolyticus in* the studied months. The results of this study are in line with the reporting of the World Health Organization and the Food and Agriculture Organization [15, 27].

In a fish meat study, the occurrence rate of *V. parahaemolyticus* in fish meat was determined according to the source (areas) of the samples. The higher occurrence rate of *V. parahaemolyticus* was recorded in the Al-Amal-City market and lower in the Al-Mahoat market. The differences between the occurrence rates in various areas and markets can be attributed to hygienic conditions and environmental factors [7]. It is noteworthy that only strains harboring the *tdh* and *trh* genes are considered pathogenic [28]. The prevalence of these pathogenic strains is generally low, as documented in various studies from different geographical locations [21, 29–32].

The occurrence of *V. parahaemolyticus* in fish meat was highest in September, the hot month. This observation is consistent with Stratev *et al*. [18] and Cruz *et al*. [29], who reported linking seawater temperature and the increased prevalence of the bacterium. Elevated abundances at high temperatures suggest that climate change affects rising seawater temperatures and higher frequencies, and the duration of heat waves may influence the abundance of *Vibrio* species.

## CONCLUSION

This study confirms the presence of *V. parahaemolyticus* in both human stool and fish meat samples in the Al-Hodeidah governorate, with an overall prevalence rate of 7.1%. Human stool samples showed a 6.7% prevalence, while sea fish meat samples exhibited a slightly higher prevalence of 7.3%. The highest human infection rates were observed in July (15.0%), while the highest fish contamination rates were recorded in September (12.0%). Among fish species, *Rastrelliger kanagurta* (Bagah) had the highest contamination rate (20.0%), whereas no isolates were found in *Dasyatis kuhlii* (Safon) and *Rachycentron canadum* (Sakalah). These findings indicate significant seasonal variations and species-specific susceptibility to *V. parahaemolyticus* contamination.

The study’s strengths lie in its comprehensive approach, analyzing both human and fish samples to provide valuable insights into cross-species transmission. The inclusion of seasonal trends offers a critical understanding of environmental influences on bacterial prevalence, contributing to public health and food safety awareness. However, the study is limited by its geographical scope, as data were collected from a single region, limiting broader generalizability. Additionally, while the sample size was adequate for statistical analysis, a larger dataset across multiple locations would enhance result reliability. The absence of molecular characterization of *V. parahaemolyticus* strains also prevents confirmation of pathogenicity markers such as *tdh* and *trh* genes.

Future research should expand to multiple coastal regions to assess broader epidemiological trends and investigate genetic determinants of virulence and antimicrobial resistance. Longitudinal studies assessing water temperature, salinity, and pollution levels could help predict seasonal outbreaks. Developing seafood safety guidelines and awareness programs will also be crucial in reducing infection risks.

These findings emphasize the importance of improving seafood handling practices, routine surveillance, and further research on transmission dynamics and mitigation strategies to enhance food safety and public health measures.

## DATA AVAILABILITY

The supplementary data can be available from the corresponding author upon a request.

## AUTHORS’ CONTRIBUTIONS

MAA, OSA, and DAA: Conceptualization and data curation. MAA, RNA, HA, OSA, and DAA: Methodology, investigation, and supervision. MAA, ADA, HA, RNA, DMAA, and AHMA: Validation. OSA, RNA, DMAA, AMA, ES, and AHMA: Formal analysis. OSA, ES, MAA, and AHMA: Resources. MAA, HA, and ADA: Writing – original draft preparation. MAA, ADA, RNA, and AHMA: Writing – review and editing. RNA, DMAA, AMA, OSA, AMA, and AHMA: Visualization. MA: Project administration and funding acquisition. All authors read and approved the final manuscript.
